# Fresh vs frozen testicular sperm for assisted reproductive technology in patients with non-obstructive azoospermia: A systematic review

**DOI:** 10.1080/2090598X.2021.1932303

**Published:** 2021-07-06

**Authors:** Medhat Amer, Emad Fakhry

**Affiliations:** a Departments of Andrology and IVF Laboratory, Adam International Hospital, Giza, Egypt; b Department of Andrology, Faculty of Medicine, Cairo University, Cairo, Egypt

**Keywords:** Azoospermia, non-obstructive azoospermia, sperm cryopreservation, testicular sperm, intracytoplasmic sperm injection

## Abstract

**Objective:**

: To review the debate about the routine use of cryopreserved testicular sperm for intracytoplasmic sperm injection (ICSI) from patients with non-obstructive azoospermia (NOA), as some authors suggest repeating sperm retrieval in such cases due to poorer ICSI results when frozen–thawed testicular sperm is used compared with fresh sperm.

**Methods:**

: A systematic literature review was performed in August 2020 using the Medical Literature Analysis and Retrieval System Online (MEDLINE), Web of Science databases and the Excerpta Medica dataBASE (EMBASE), and we included 26 studies that were considered eligible for this systematic review.

**Results:**

: In all, 1189 publications were screened and 26 articles were included in the systematic review. Three meta-analysis reviews were included and they all concluded that the use of fresh and frozen sperms for ICSI from patients with NOA showed comparable fertilisation and pregnancy rates.

**Conclusion:**

: The use of frozen testicular sperm from men with NOA results in fertilisation and clinical pregnancy rates similar to those of fresh sperm. This may encourage fertility centres to use frozen testicular sperm samples, as this policy has certain advantages that would help with organising their workflow.

**Abbreviations**: CPR: clinical pregnancy rate; 2PN%: two pronuclei % fertilisation rate; ICSI: intracytoplasmic sperm injection; NOA: non-obstructive azoospermia; OA, obstructive azoospermia; SCO: Sertoli cell-only syndrome; (micro-)TESE: (microsurgical) testicular sperm extraction

## Introduction

Azoospermia is defined by a complete absence of sperm in the ejaculate after centrifugation of two semen specimens and affects about 1–2% of males and 10–15% of the infertile males [1,2].

Obstructive azoospermia (OA) is the absence of spermatozoa in the ejaculate secondary to a transport failure between the testis and urethra [3]. While two-thirds of azoospermic cases are categorised as non-obstructive azoospermia (NOA) caused by spermatogenic failure, which means failure to produce sperm in the testes, with a spectrum of various causes of intrinsic testicular impairment, but fortunately focal areas of sperm production can be found in some of these men with NOA [4].

An isolated diagnostic testicular biopsy should rarely be indicated, as it will not provide definitive proof of whether sperm will be found during sperm retrieval, particularly in Sertoli cell-only syndrome (SCO) and maturation arrest cases [5–10]. Histopathological evaluation by removal of rare spermatogenesis foci may jeopardise future retrieval attempts [10]. So diagnostic biopsies are indicated if the differential diagnosis between OA and NOA cannot be established based on clinical and endocrine parameters, and also for screening for carcinoma *in situ* in patients with azoospermia [11].

A suitable treatment for NOA is microsurgical testicular sperm extraction (micro-TESE) followed by intracytoplasmic sperm injection (ICSI). A testicular biopsy can be performed on the day of oocyte retrieval and fresh sperm can be used to fertilise the oocytes [12].

For those cases with anticipated difficult sperm retrieval, it is better to start testicular sperm retrieval at least 8 h before ovum retrieval to avoid post-maturity oocyte damage [3]. However, this can cause scheduling conflicts (operating room availability and the urologist may change his time schedule). Another option is to perform the surgical sperm retrieval independent of the ovum retrieval day and freeze the testicular sperm. An advantage of this is that the couple will know in advance that testicular sperm is available and therefore not worry about the possibility of futile ovarian stimulation and financial loss. Cryopreserved sperm also allows the embryologist to know whether viable spermatozoa are available for ICSI before oocyte retrieval. Cryopreservation of testicular sperm is generally recommended for fear of future failure in order to obtain suitable spermatozoa for ICSI in such patients [11].

Some authors suggest repeating the sperm retrieval in such cases due to poorer ICSI results when frozen–thawed testicular sperm samples are used compared with fresh sperm samples, as they are convinced that during the process of freezing and thawing spermatozoa are subjected to a series of drastic changes in their environment. Phase transitions of the lipids in the plasma membrane may impair the function of membrane proteins that are needed for ion metabolism and structural integrity. Freezing may also lead to extracellular ice nucleation producing osmotic changes with efflux of water from the cells, with loss of stability of the lipid bilayer. Further consequences may also include denaturation of proteins and structural deformation of the cell organelles [13,14].

The aim of the present systematic review was to review the debate about the routine use of cryopreserved testicular sperm in ICSI in patients with NOA, as some authors suggest repeating the sperm retrieval in such cases due to poorer ICSI results when frozen–thawed testicular sperm samples are used compared with fresh sperm samples.

## Methods

**Figure 1.**
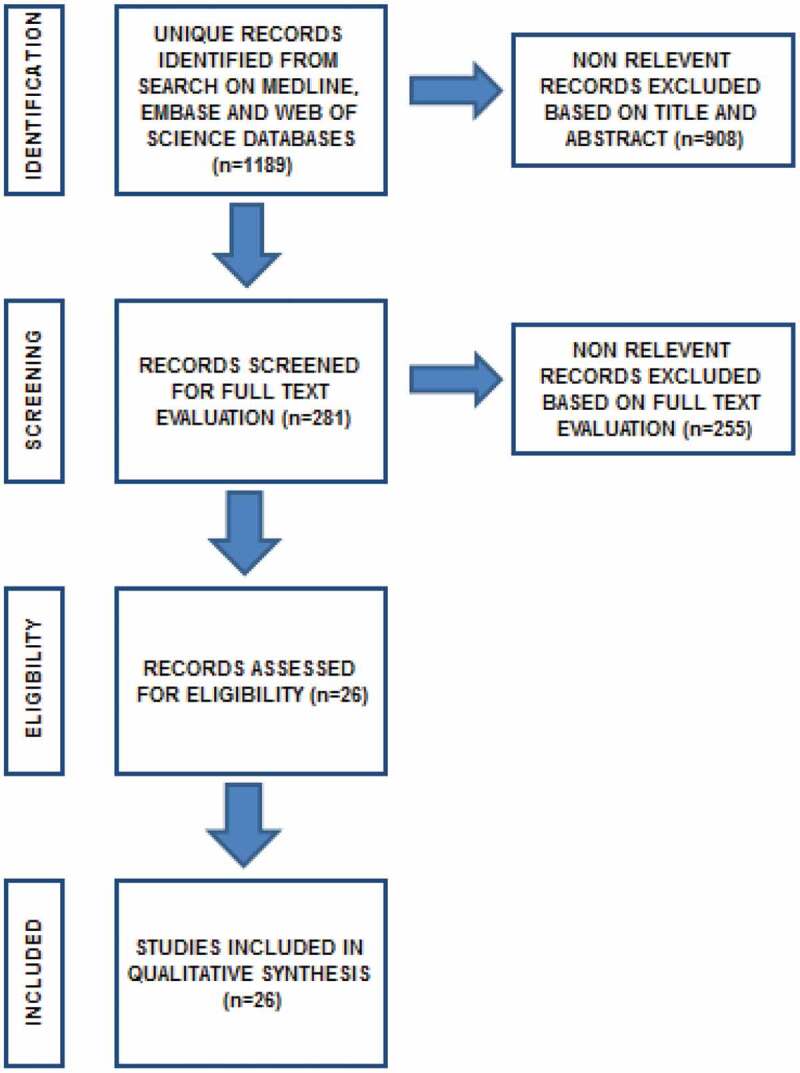
Flow diagram of the search results

A systematic literature review was performed in August 2020 using the Medical Literature Analysis and Retrieval System Online (MEDLINE), Web of Science databases, and the Excerpta Medica dataBASE (EMBASE). Review articles, congress abstracts and editorials were excluded. Search terms included ‘non-obstructive azoospermia’ in combination with the term ‘cryopreservation’ OR in combination with the term ‘intracytoplasmic sperm injection’. The search was limited to the English literature. References cited in selected articles and in review articles retrieved in the search were used to identify other studies and articles that were not included in the initial searches. We included the articles that provided the highest level of evidence. The systematic review was performed following the Preferred Reporting Items for Systematic Reviews and Meta-Analyses (PRISMA) guidelines [15] (Figure 1).

## Results

In all, 1189 publications were screened and 26 of them were included in this systematic review based on our inclusion criteria and were considered eligible for this study. Duplicate studies and abstracts were excluded. Only full-text articles in the English language were included. Non-relevant records were excluded based on title and abstract (*n* = 908). Non-relevant records were excluded based on full-text evaluation (*n* = 255). Three meta-analysis reviews were included and they all concluded that the use of fresh and frozen sperm samples in ICSI in patients with NOA showed comparable fertilisation and pregnancy rates.

## Discussion

Cryopreservation of testicular sperm is routinely recommended for fear of future failure to obtain motile spermatozoa in NOA cases. Some authors suggest repeating the sperm retrieval in such cases due to poorer ICSI results when frozen–thawed testicular sperm samples are used compared with fresh sperm samples. The present systematic review focussed on this debate. A systematic literature review was performed in August 2020 using MEDLINE, Web of Science databases and EMBASE. We screened 1189 publications and 26 articles and three meta-analysis reviews were included.

**Figure 2.**
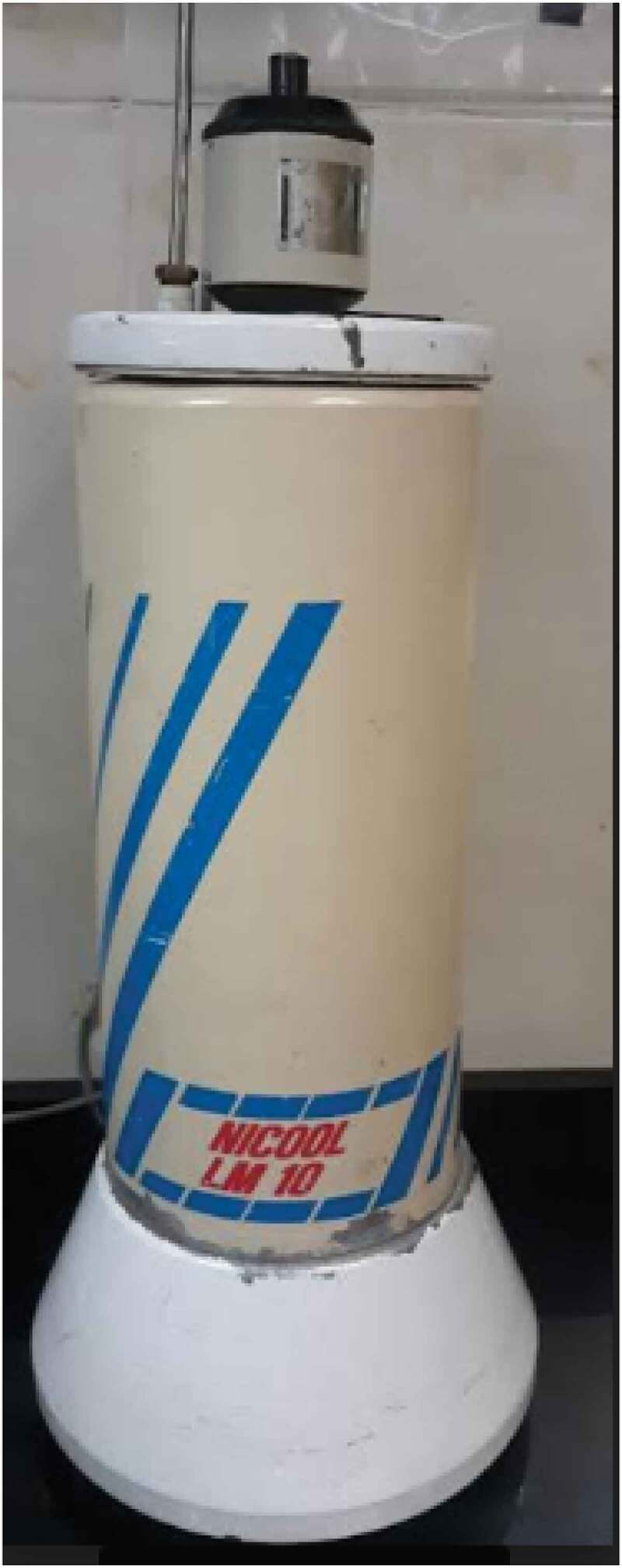
Nicool LM10 semi-programmable freezer

The success of testicular sperm cryopreservation and their further use in ICSI was first reported in the 1990s. We may refer to Table 1 for a comparison of ICSI outcomes between fresh and frozen testicular sperm from patients with NOA to glean confidence in the frozen sperm results in ICSI [16–37]. Table 1 also shows that the cryopreservation protocol varied between the laboratories in the different studies. It is obvious that there are two choices for testicular sperm preservation, freezing the sperm-containing suspensions or the testicular tissue sample, and both options are used in the selected studies. In most of the studies dealing with sperm cryopreservation, the most commonly used method was freezing in liquid nitrogen vapour for 15–30 min without the use of any device (Figure 2) that would control the freezing slope, which was previously documented as being crucial for the healthiness of the sperm after thawing [38].

**Table 1.** Comparison of ICSI cycles using fresh vs frozen–thawed surgical retrieved testicular sperm

Publication	Freezing methodology	Compared with	Frozen NOA cycles, n	Fresh cycles, n	Fertilisation rate (2 PN%), %		Good embryo, %		CPR/ET, %		IR, %		LBR/ET, %		Significant results
					Frozen sperm	Fresh sperm	Frozen sperm	Fresh sperm	Frozen sperm	Fresh sperm	Frozen sperm	Fresh sperm	Frozen sperm	Fresh sperm	
**Friedler *et al*., 1997 [**16**]**	Testicular tissue, test yolk buffer (Irvine) then nitrogen vapour chamber (–10°C/min) for 20 min	Fresh testicular sperm from NOA cases	14	*25*	43.5	*60.7*	77.1	*77.4*	21.4	*24.0*	10.8	*8.8*	9.1	*21.7*	No difference in 2 PN%, embryo quality, CPR, IR and LBR between the two studied groups
**Ben-Yosef *et al*., 1999 [**17**]**	Testicular sperm suspension, test yolk buffer (Irvine) then Nicool Semi-programmable freezer (two steps) (Figure 2)	Fresh testicular sperm from NOA cases	42	*15*	55.5	*53.8*	74.6	*66.1*	21.4	*26.7*	10.7	*12.5*	16.7	*20.0*	No difference in 2 PN%, embryo quality, CPR, IR and LBR between the two studied groups
**Habermann *et al*., 2000 [**18**]**	Testicular sperm suspension, TEST-citrate-yolk buffer with 12% glycerol then two step controlled cooling	Fresh testicular sperm from NOA (OA cases were also compared)	9	*3*	55.7	*51.6*			66.7	*33.3*	24.1	*12.5*	33.3	*33.3*	No difference in 2 PN%, CPR, IR and LBR between the two studied groups
**Fukunaga *et al*., 2001 [**19**]**	Testicular sperm suspension, test yolk buffer (Irvine) then 15 min in LNV	Fresh testicular sperm from NOA cases (OA cases were also compared)	11	*9*					9.1	*33.3*					No difference in CPR between the two studied groups but the frozen sperm CPR was significantly lower in NOA than OA cases
**Friedler *et al*., 2002 [**20**]**	Testicular sperm suspension, test yolk buffer (Irvine) then nitrogen vapour-containing chamber (–10°C/min)	Fresh testicular sperm from NOA cases (OA cases were also compared)	63	*65*	51.0	*51.0*			25.8	*27.1*	17.4	*12.7*	23.8	*24.6*	No difference in 2 PN%, CPR, IR and LBR between the two studied groups
**Sousa *et al*., 2002 [**21**]**	Testicular sperm suspension, sperm freezing medium (Medicult) then 10–15 min in LNV	Fresh testicular sperm from NOA cases (spermatid injection cases were also studied)	37	*50*	60.0	*68.2*	84.2	*86.8*	24.3	*34.0*					No difference in 2 PN%, embryo quality, CPR between the two studied groups
**Verheyen *et al*., 2004 [**22**]**	Testicular sperm suspension, test yolk buffer (Irvine) then 16 min in LNV (two steps)	Fresh testicular sperm from NOA cases	42	*44*	59.3	*58.0*			18.7	*17.1*	7.4	*7.6*			No difference in 2 PN%, CPR and IR between the 2 studied groups
**Hauser *et al*., 2005 [**23**]**	Testicular tissue, test yolk buffer (Irvine) then Nicool semi-programmable freezer (two steps) (Figure 2)	Fresh testicular sperm from NOA cases versus own frozen sperms	13	*13*	35.2	*41.8*	66.7	*61.5*	15.4	*18.2*	5.9	*10.5*	15.4	*18.2*	No difference in 2 PN%, embryo quality, CPR and IR between the two studied groups
**Wu *et al*., 2005 [**24**]**	Testicular tissue, test yolk buffer (Irvine) then 15 min in LNV	Fresh testicular sperm from NOA and OA cases	24	*6*	65.8	*74.5*	53.8	*52.9*	62.5	*33.3*	25.0	*15.8*	41.7	*33.3*	Differences in CPR, IR and LBR between the frozen (better) and the fresh group
**Konc *et al*., 2008 [**25**]**	Testicular tissue, sperm freeze (FertiPro) then 15 min in LNV	Fresh testicular sperm from NOA cases	93	*64*	66.4	*63.6*			23.7	*31.3*	6.6	*10.8*	18.3	*15.6*	No difference in 2 PN%, CPR, IR and LBR between the two studied groups
**Akarsu *et al*., 2009 [**26**]**	Sperm freezing protocol was not mentioned	Fresh testicular sperm from NOA cases	2	*4*	19.4	*57.8*			0.0	*75.0*			0.0	*75.0*	No difference in 2 PN% and CPR between the two studied groups
**Kalsi *et al*. 2010 [**27**]**	Testicular sperm suspension, sperm freezing med. (unspecified) then 15 min in LNV	Fresh testicular sperm from NOA cases (OA cases were also compared)	7	*41*	63.4	*50.8*			57.1	*36.6*			57.1	*31.7*	A difference in CPR and LBR in favour of the frozen sperm group
**Karacan *et al*. 2013 [**28**]**	Testicular sperm suspension, Sperm Freeze (FertiPro) then 30 min in LNV	Fresh testicular sperm from NOA cases (OA cases were also compared)	84	*99*	64.7	*67.2*			23.8	*29.2*	12.3	*12.6*	21.4	*27.2*	No difference in 2 PN%, CPR, IR and LBR between the two studied groups
**Abdel Raheem *et al*. 2013 [**29**]**	Testicular tissue, sperm freezing med. (Medicult) then controlled rate freezer (two steps)	Fresh testicular sperm from NOA cases (OA cases were also compared)	46	*31*	45.7	*56.0*	85.0	*88.0*	34.8	*29.1*			19.6	*19.4*	No difference in 2 PN%, embryo quality, CPR and LBR between the two studied groups
**Tavukcuoglu *et al*. 2013 [**30**]**	Testicular tissue, sperm freezing med. (Vitrolife) then nitrogen vapour-containing Styrofoam box 20–30 min	Fresh testicular sperm from NOA cases	39	*43*	46.8	*44.8*	51.3	*58.1*	43.6	*44.2*			30.8	*37.2*	No difference in 2 PN%, embryo quality, CPR and LBR between the two studied groups
**Madureira *et al*. 2014 [**31**]**	Testicular sperm suspension, sperm freezing med. (Origio) then LNV	Fresh testicular sperm from NOA cases (Non-mosaic KF cases)	17	*20*	43.2	*60.0*	55.4	*75.6*	23.5	*60.0*	17.2	*34.1*	23.5	*50.0*	Differences in 2 PN%, embryo quality and CPR in favour of the fresh sperm group
**Hessel *et al*. 2015 [**32**]**	Testicular sperm suspension, test yolk buffer (Irvine) then 5–10 min in LNV	Fresh testicular sperm from NOA cases	600	*145*					22.3	*21.3*			21.0	*20.6*	No difference in CPR and LBR between the two studied groups
**Park *et al*. 2015 [**33**]**	Testicular tissue, cryosperm med. (Medicult) then computerised cell freezer (CryoMagic-Plus; two steps)	Fresh testicular sperm from NOA cases	49	*61*	68.5	*59.6*	66.3	*64.5*	38.1	*22.0*	56.3	*21.3*	4.8	*12.2*	A difference in IR in favour of the frozen sperm group
**Schachter-Safrai *et al*. 2017 [**34**]**	Testicular sperm suspension, sperm freezing med. (Origio) then nitrogen vapour in a plastic freezing box (–1°C/min)	Fresh testicular sperm from NOA cases (cryptozoospermic cases were also compared)	48	*22*	49.0	*52.1*			18.8	*18.2*	12.2	*7.3*	18.8	*4.5*	No difference in 2 PN%, CPR, LBR and IR between the two studied groups
**Okuyama *et al*. 2018 [**35**]**	Testicular sperm suspension, sperm freeze (Medi-con) then 15 min in LNV	Fresh testicular sperm from NOA cases (Fresh oocytes) (OA cases and cryptozoospermic cases were also compared)	78	*71*	52.6	*59.4*			34.3	*49.3*			12.5	*31.5*	A difference in LBR in favour of the fresh sperm group
**Falah 2019 [**36**]**	Testicular sperm suspension, sperm freeze (FertiPro) then 15 min in LNV	Fresh testicular sperm from NOA cases (OA cases were also compared)	36	*32*	36.0	*37.0*			14.3	*20.0*			3.6	*4.0*	No difference in 2 PN%, CPR and LBR between the two studied groups
**Zhang *et al*. 2021 [**37**]**	Testicular sperm suspension, sperm freezing med. (Vitrolife) then 30 min in LNV	Fresh testicular sperm from NOA cases	110	234	44.2	*47.6*	53.7	*52.1*	40.5	*52.3*	33.5	*36.2*	34.5	*48.7*	Differences in CPR and LBR in favour of the fresh sperm group

ET: embryo transfer; IR, implantation rate; LBR: live-birth rate; LNV: liquid nitrogen vapour; med.: medium.

Some fertility specialists are convinced that during the process of freezing and thawing, spermatozoa are harmed and so they recommend repeating the sperm retrieval procedure in such cases to avoid poorer ICSI results when frozen–thawed testicular sperm samples are used. A potential risk to be considered is testicular sperm loss after freezing and thawing, as survival of frozen–thawed samples is not uniform in all centres (20–90%), while repeating the sperm retrieval procedure cannot be done before 3 [39] or 6 months [40].

So to settle the debate about the success rates of fresh vs frozen testicular sperm use, three meta-analysis reviews were conducted. They all concluded that fresh and frozen sperms in ICSI from patients with NOA showed comparable fertilisation and pregnancy rates.

The first meta-analysis review article by Nicopoullos *et al*. [41] in 2004 from the Chelsea and Westminster Hospital, London, compared the use of fresh vs frozen testicular sperm ICSI in men with azoospermia (OA and NOA). They reviewed the available data to determine if frozen testicular sperm samples were associated with decreased fertilisation and pregnancy rates. The authors identified a total of 17 studies and data were analysed from 1476 cycles. No difference in fertilisation, clinical pregnancy rate (CPR) and ongoing pregnancy rate was noted when the testicular cycles were analysed separately, but implantation was significantly impaired using frozen–thawed sperm.

In the second meta-analysis review article by Ohlander *et al*. [42] in 2014 from the University of Illinois, Chicago, a total of 224 studies were identified, 11 of which met criteria for inclusion. Data were analysed from 274 cycles with fresh and 299 cycles with frozen testicular sperm (in only men with NOA to be more specific than the first meta-analysis). Fertilisation rates (two pronuclei % [2 PN%]) were similar when comparing fresh vs frozen sperm (52.9% and 54%, respectively). In addition, the CPR was similar when comparing fresh vs frozen sperm (28.7% and 28.1%, respectively).

In the third meta-analysis review article by Yu *et al*. [43] in 2018 from Huazhong University in China, a total of 997 studies were reviewed, of which 17 met the inclusion criteria. Data were analysed from 1261 cycles. Fertilisation, good embryo, CPR, implantation rate and live-birth rate were similar when comparing fresh vs frozen sperm (Table 2) [41–43].

**Table 2.** The three meta-analyses studying the effects of testicular sperm freezing on the ICSI outcome in NOA

Meta-analysis	Revised years	Revised studies, *n*	ICSI cycles, *n*	Significant results
Nicopoullos *et al*., 2004 [41]	1995–2002	17	1476	No difference in 2 PN%, CPR and ongoing pregnancy rate between the two groups, but implantation rate was higher in the fresh sperm group.
Ohlander *et al*., 2014 [42]	Before 2012	11	573	No difference in 2 PN% and CPR between the two groups.
Yu *et al*., 2018 [43]	1997–2017	17	1184	No difference in 2 PN%, embryo quality, CPR, implantation rate and live-birth rate between the studied groups.

Surgical retrieval of rare sperms can be achieved in clinical practice. Conventional cryopreservation cannot help those patients due to its technical limitations. More complicated technologies have been developed over the years to manage this situation. A number of devices have been suggested to cryopreserve rare spermatozoa like empty zona, Cryolock, Cell Sleepers, Petri dishes, the novel sperm vitrification device (SpermVD) and other biological or non-biological devices. None of these have achieved widespread use due to technical requirements and cost concerns [44].

## Conclusion

Use of frozen testicular sperm from men with NOA results in fertilisation rates similar to that of fresh sperm samples. In addition, the CPR was found to be similar when comparing fresh vs frozen sperm use in that group of patients. This may encourage fertility centres to use frozen testicular sperm samples, as this policy has certain advantages that would help organising the workflow.

## Summary and key points

Significant progress has been made over the past few years in our understanding of male infertility. This understanding, as well as rapid technological progress, has played a great role in managing males with azoospermia. In view of these findings, sperm cryopreservation has to be considered in every surgical sperm retrieval operation to guarantee the best use of those valuable sperms. Patients with azoospermia need to be reassured that frozen–thawed viable spermatozoa are as good as the fresh ones, and they should be adequately counselled before any surgical procedure (Table 3 for proper patient counselling [3,39,40,45,46]).

**Table 3.** A comparison between the different policies while dealing with a NOA case

Policy	Indications	Advantages	Disadvantages	Special counselling
**A. TESE on the day of oocyte retrieval OPU (with sperm cryopreservation of the remaining samples)**	1. Expected positive cases: previous positive TESE, cryptozoospermic ejaculate or virtual azoospermia (pervious presence of spermatozoa in the ejaculate), favourable previous histopathological diagnosis such as hypospermatogenesis, maturation arrest at spermatid, mixed patterns with normal spermatogenesis. 2. Expected difficulty with limited possibility to repeat TESE in the future: severe gonadal failure, e.g. previous genetic or histopathological diagnosis of Klinefelter syndrome or small testes where repeating biopsy seems improbable. 3. Expected low sperm number with difficulty in freezing (redo-patients and patients with documented deletions of the AZFb region) if the couple accept the high possibility of sperm retrieval failure [3].	1. The use of fresh testicular sperm sample with no fear of losing sperm motility after freezing. 2. Avoidance of repeating the TESE procedure if no motile spermatozoa were found in the frozen-thawed sample on the day of ICSI in a small testicular size male. 3. The possibility of use of very limited number of sperms with poor or no motility that are not suitable for freezing.	1. Pointless ovarian stimulation, risk of hyperstimulation, financial burden if no spermatozoa were retrieved. 2. The TESE procedure must be scheduled on the day of OPU, which is not practical in a busy IVF laboratory or for the surgeon. 3. Risk of *in vitro* post maturity of oocytes associated with low fertilisation rate and poor embryo quality after ICSI in difficult prolonged sperm search [45].	Risk of finding no sperms is great, so the couple should accept this fact and according to their preference, oocyte retrieval can be cancelled or the oocytes are collected and vitrified for future hope: Possibility of finding sperm in the future in a redo TESE, spontaneously in extended ejaculated sperm pellet analysis, hormonal treatment or future advances in NOA management (spermatid injection, *in vitro* maturation). TESE before oocyte retrieval for expected difficult cases should preferably be scheduled 4–8 h before ovum pick-up to allow more time to extract and collect sufficient normal motile testicular sperm for injection of all available oocytes [45].
**B. Diagnostic TESE (with sperm cryopreservation followed later by female preparation for ICSI)**	1. Normal or moderate size testicle and if the couple accept the minor risk of testicular sperm loss after freezing and thawing. 2. Expected negative cases for sperms: previous negative TESE, unfavourable previous histopathological diagnosis such as SCO, maturation arrest at primary spermatocyte or tubular hyalinisation, if the couple accept the possibility that sperm freezing may fail in cases of very few sperms or totally immotile sperms.	1. Avoidance of unnecessary ovarian stimulation, risk of ovarian hyperstimulation also the ICSI cycle can be started while knowing that the couple have an opportunity to achieve pregnancy. Freedom to proceed into an ICSI cycle at any time appropriate for the couple. 2. Obvious practicality in a busy IVF laboratory: full, exhaustive and unpressured examination of the testicular tissue can be accomplished when extracting testicular tissue on a day completely different from oocyte retrieval. 3. Testicular sperm extraction may be performed at the same time of: diagnostic biopsy, backup in reconstructive procedures or during varicocelectomy [46]. 4. Avoidance of repetition of testicular biopsy as multiple vials or straws of the frozen-thawed sample are stored, the patients may not require any further operative procedures [46]. 5. No procedures on the same day of OPU would prevent the couple’s exposure to physical, psychological or financial stress especially if no sperms were retrieved, as the procedure is scheduled electively before female stimulation.	1. Risk of testicular sperm loss after freezing and thawing. Survival of frozen-thawed samples is not uniform in all centres (20–90%), while repeating the sperm retrieval procedure cannot be done before 3 [39] or 6 months [40]. 2. Pregnancy rate is variable from centre to centre with frozen–thawed sperm samples.	There is always a possibility that the TESE procedure be repeated on the occasion of finding no motile spermatozoa in the frozen-thawed samples, so the husband must be present in the centre and should be prepared for a redo TESE. This possibility is higher in patients with very low sperm count and viability.

AZF: Azoospermia factor; IVF: *in vitro* fertilisation; OPU: ovum pickup.
